# Asian Attitudes and Perceptions Toward Hospital-At-Home: A Cross-Sectional Study

**DOI:** 10.3389/fpubh.2021.704465

**Published:** 2021-07-23

**Authors:** Yi Feng Lai, Yee Wei Lim, Win Sen Kuan, Joel Goh, John Tshon Yit Soong, Shefaly Shorey, Stephanie Q. Ko

**Affiliations:** ^1^Ministry of Health (MOH) Office for Healthcare Transformation, Singapore, Singapore; ^2^Department of Pharmacy, Alexandra Hospital, Singapore, Singapore; ^3^Department of Pharmacy, National University of Singapore, Singapore, Singapore; ^4^School of Public Health, University of Illinois at Chicago, Chicago, IL, United States; ^5^Department of Medicine, Yong Loo Lin School of Medicine, National University of Singapore, Singapore, Singapore; ^6^Emergency Medicine Department, National University Hospital, Singapore, Singapore; ^7^National University of Singapore (NUS) Business School, National University of Singapore, Singapore, Singapore; ^8^Global Asia Institute, National University of Singapore, Singapore, Singapore; ^9^Harvard Business School, Harvard University, Boston, MA, United States; ^10^Department of Medicine, National University Hospital, Singapore, Singapore; ^11^Alice Lee Centre for Nursing Studies, Yong Loo Lin School of Medicine, National University of Singapore, Singapore, Singapore

**Keywords:** home care services, hospital-based, attitude to health, perception, hospital care, Hospital at Home

## Abstract

**Introduction:** Hospital-at-Home (HaH) programmes are well-established in Australia, Europe, and the United States. However, there is limited experience in Asia, where the hospital is traditionally seen as a safe and trusted space for healing. This cross-sectional study aimed to explore attitudes and perceptions among patients and caregivers in Singapore toward this care model.

**Methods:** A quantitative study design was adopted to collect data among patients and their caregivers from medical wards within two acute hospitals in Singapore. Using a series of closed-ended and open-ended questions, the investigator-administered survey aimed to explore barriers and facilitators determining patients' and caregivers' responses. The study questionnaire was pretested and validated. Data were summarised using descriptive statistics, and logistic regression was performed to determine key factors influencing patients' decisions to enrol in such programmes.

**Results:** Survey responses were collected from 120 participants (101 patients, 19 caregivers; response rate: 76%), of which 87 respondents (72.5%) expressed willingness to try HaH if offered. Many respondents valued non-quantifiable programme benefits, including perceived gains in quality of life. Among them, reasons cited for acceptance included preference for the comfort of their home environment, presence of family members, and confidence toward remote monitoring modalities. Among respondents who were unwilling to accept HaH, a common reason indicated was stronger confidence toward hospital care.

**Discussion:** Most patients surveyed were open to having acute care delivered in their home environment, and concerns expressed may largely be addressed by operational considerations. The findings provide useful insights toward the planning of HaH programmes in Singapore.

## Introduction

Hospital-at-Home (HaH) is a care model which provides hospital-level acute inpatient services in the comfort of the patient's home. An early version was described by Leff and Burton in 1996 as a “care option that could help certain patients avoid inpatient hospitalization altogether” (1). In HaH care model, remote monitoring and telecommunication technologies can be used to enable the delivery of hospital-level care, which include physician consultations, medication administration, nursing, and therapy services, clinical diagnostics, and investigations, etc.

HaH models have been implemented in countries such as Australia, the United States, Canada, the United Kingdom, and Spain (2). This model of care was implemented as a possible solution to various issues associated with delivery healthcare in hospital. These include increasing rates of nosocomial infections and the rising prevalence of patients with multiple comorbidities who require frequent readmissions (3–5). Studies have shown that the implementation of HaH can result in improved clinical outcomes, in terms of a shorter length of stay and reduced readmission rates (6, 7). It may also bring about greater patient and caregiver satisfaction, along with cost savings for both the patients and the healthcare system (7–9). Mortality rates have been shown to be equivalent to that of patients warded in hospitals, highlighting the safety of this care model (9, 10). HaH has been found to reduce mental stress and adverse hospital events such as functional decline, incontinence, and delirium experienced by elderly who undergo acute hospital admissions (5).

The rapidly ageing population in Singapore has led to growing healthcare expenditure, insufficient hospital beds, and a shortage of clinicians (6). In response to this trend, the Ministry of Health has outlined a shift in focus from hospital to community care. Aligning with this direction, HaH presents a potentially scalable alternative that can lower nationwide healthcare expenditures, alleviate care provider shortages through the use of technology and partnership with community service providers to enhance productivity, and reduce demand for inpatient beds. The necessity of implementing a more adaptable model of care has been exacerbated by the COVID-19 pandemic, which has placed huge strains on local and global healthcare systems.

There have been recent trends that set a favourable landscape for HaH, such as the rising acceptance of telehealth video consultations as an alternative to physical consults, the popularisation of transitional home care services, and the growth of private home care providers. However, because hospital services have historically been confined to the physical walls of a hospital, the public perception of HaH has yet to be well-established. Acceptance of HaH may differ from those of other countries, due to differing cultural views, financing norms and hospital cost structures, and there may be uncertainty in cost savings gained through HaH and patients' perceptions of the safety of HaH (10–12).

This study aimed to elicit attitudes and perceptions among hospitalised patients and their caregivers toward HaH in Singapore, so as to determine the prospects of developing such care models in the future.

## Methods

### Settings

This study was conducted at two hospitals in Singapore which currently do not have a HaH programme. National University Hospital (NUH) is an academic medical centre and major referral centre with over fifty medical, surgical, and dental specialties. Each year, the 1,239-bed hospital attends to more than one million patients. It serves as a clinical training centre and academic research centre for the medical and dental faculties of the National University of Singapore. Alexandra Hospital (AH) is a general hospital with 326 beds providing comprehensive care to residents in the southwestern region of Singapore, and provides care spanning acute, sub-acute, and rehabilitative settings.

### Study Design

A cross-sectional quantitative study was conducted among patients and caregivers at both NUH and AH, from June to August 2020.

Study inclusion criteria were: (1) Patients admitted to NUH and their caregivers during the study period; or (2) Patients admitted to AH medical wards and their caregivers during the study period. All participants were 21 years or older. For this study, a caregiver was defined as the main spokesperson or family member of the patient. Caregivers were approached in the wards during visitation hours if patients were unable to respond, or if they specifically requested the investigators to speak to them instead. The study excluded patients or caregivers who were cognitively impaired and unable to provide informed consent.

Eligible patients and caregivers were recruited by a team of five trained researchers to discuss the study and obtain informed consent at both hospital sites. During the consent-taking process, patients and caregivers were also asked if they would be agreeable for the investigators to access their clinical documentation for additional data collection and be contacted for further interviews if required.

### Survey Design

The questionnaire developed by the study investigators aimed to collect general information of their views on receiving inpatient care in a home setting. The survey explored the following domains: (a) background and demographic information, (b) healthcare professionals and procedures encountered during an inpatient stay, (c) perspectives if each care element were delivered at home instead (including home visits, home therapy, remote monitoring, and communication equipment), (d) general views about receiving care at home, and (e) views on the financing models for HaH. Patients and caregivers were assumed to be unfamiliar with HaH care models as this was not the standard of care in Singapore.

The study questionnaire was designed and pre-tested by the study team before actual data collection. Face and content validity were examined through cognitive debriefing, while construct validity, reliability, and internal consistency were examined using empirical data and hypothesis testing through Cronbach's alpha and intraclass correlation (13). The test-retest reliability of the questionnaire was found to be high, with an intraclass correlation of above 0.99. Cronbach's alpha in measurements of various domains were found to be within the satisfactory range of 0.69–0.87.

To determine the sample size for this study, we defined the primary outcome as the proportion of patients who would accept HaH care if it were offered. We then calculated the number of samples required using confidence level of 95% and margin of error of 10%, assuming that there would be an unequal proportion of patients who would accept HaH care compared to those who would reject HaH care. This calculation resulted in a minimum sample size of 97. Recognising a potential situation that the survey would not be carried out with patients exclusively, the study team targeted to complete 120 surveys, with at least 97 of them conducted with patients directly, and not with their caregivers or spokespersons.

### Data Analysis

Patient demographics and characteristics were summarised using descriptive statistics – frequency tabulations for categorical variables and summary parameters for continuous variables (means and standard deviation). The primary outcome measure was the proportion of respondents who indicated acceptance of HaH. Secondary exploratory analyses examined the association between the primary outcome measure and selected predictive variables (including patients' age, gender, race, command of selected languages, citizenship, Barthel Index, ward location, employment status, housing type, marital status, presence of domestic helper, education, household per capita income, self-rated overall health) through logistic regression. We hypothesised that most of these demographic variables could directly or indirectly influence the decision to accept or reject the programme among our patients. Data management and analyses were performed with STATA version 13.1 (StataCorp LP, College Station, TX).

### Ethics Approval

This study was approved by the National Healthcare Group Domain Specific Review Board (Ref: 2020/00127).

## Results

From June 2020 to July 2020, a total of 158 patients were screened to be eligible, and 101 patients responded to the survey in person while 19 caregivers responded on behalf of the patient (response rate = 75.9%). Out of the 120 respondents who completed the survey, 92 (76.7%) respondents provided consent to access their clinical and demographic information, which were used in the study's logistic regression, from their electronic medical records (EMR).

The mean age of the patients surveyed was 53.6 years. Demographic and clinical characteristics of consenting patients are summarised in Table 1.

**Table 1:** Demographic and clinical characteristics of patients surveyed.

**Demographic and clinical variables (from EMR)**	**Observations (*n* = 92)**
Patient age (years), mean (SD)	53.6 (15.3)
Patient gender, *n* (%)	
Female	40 (43.6)
Patient race, *n* (%)	
Chinese	52 (56.5)
Malay	15 (16.3)
Indian	18 (19.666)
Others	7 (7.6)
Patient ward class, *n* (%)	
A/B1 (less government subsidy)	4 (4.3)
B2/C (more government subsidy)	88 (95.777)
Length of hospital stay (days), mean (SD)	4.3403403 (4.0)
Primary diagnosis, *n* (%)	
Infectious diseases	17 (18.555)
Gastrointestinal diseases	11 (202012.0)
Neurological diseases	9 (9.888)
Arthritis, degenerative joint diseases	7 (7.6)
Fall, functional deconditioning	6 (6.5)
Others	42 (45.777)
**Non-clinical demographic variables (from survey)**	**Observations (*****n*** **=** **120)**
Employment status, *n* (%)	
Unemployed	20 (16.777)
Employed part time	16 (13.3)
Employed full time	44 (36.777)
Self employed	7 (5.8)
Retired	33 (27.5)
Housing type, *n* (%)	
HDB 1–2 room flat	11 (9.222)
HDB 3–4 room flat	63 (52.5)
HDB 5 room flat/Executive condominium	31 (25.8)
Private condominium	12 (10.0)
Private landed property	3 (2.5)
Education, *n* (%)	
No formal education	8 (6.777)
Primary	16 (13.)
Secondary	55 (45.8)
Pre-university	4 (3.)
Diploma	16 (13.)
Degree and above	21 (17.5)
Household per capita income, *n* (%)	
No income	25 (20.8)
< $1,000	78 (65.)
$1,000–3,000	14 (11.7)
$3,000–5,000	3 (2.5)
Marital status, *n* (%)	
Single, never married	25 (20.8)
Married or domestic partnership	78 (65.)
Widowed	14 (11.7)
Divorced or separated	3 (2.5)
Presence of dhdhdomestic helper, *n* (%)	
Yes	25 (20.8)
No	95 (79.222)

**Figure 1:**
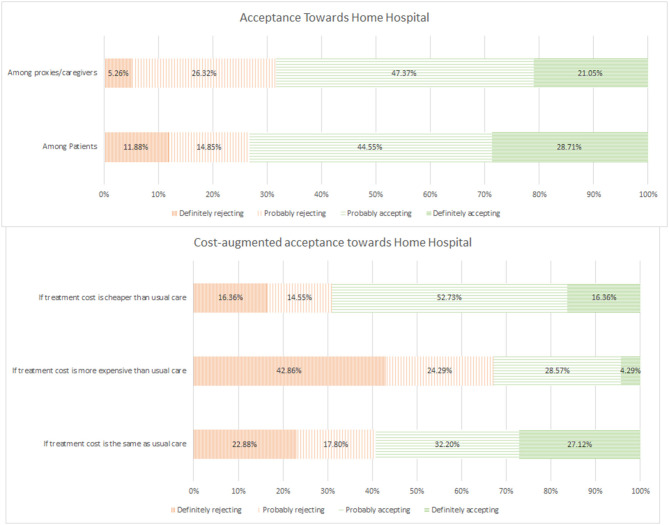
Acceptance toward home hospital.

Among the 120 respondents, 87 (72.5%) expressed acceptance toward the novel care model (Figure 1); 29 (28.7%) and 45 (44.6%) patients indicated their definite and probable willingness to participate in a HaH programme, respectively, and (21.1%) and 9 (47.4%) caregivers reported definite and probable willingness to allow their loved ones undergo HaH, respectively. Commonly cited reasons for acceptance included perceived comfort at home, being surrounded by family members, and perceived confidence in remote monitoring as well as the presence of hospital providers helming the care process. On the other hand, frequently cited reasons for rejection included preference for care in hospitals, lack of confidence in technology or care modality, and doubts regarding the ability of hospital providers to deliver comparable care in the home environment.

More than half of the respondents surveyed were open to consider home hospitalisation as an alternative to their current inpatient hospitalisation (Table 2). HaH acceptance seemed to be primarily driven by perceived comfort (85% of accepting patients) and the company of loved ones (85% of accepting patients). Many were not deterred by potential risks and were comfortable with remote monitoring of vital signs (82% of accepting patients) and had high level of confidence in remote care delivery (81% of accepting patients). There was also a high degree of acceptance for tests and care procedures to be carried out at home.

**Table 2:** Summary of survey findings.

**Survey findings**	**Observations (*n* = 120)**
**Service acceptance among respondents agreeing to home hospital care, %**	
Transferring to hospital for diagnostic tests/scans	98
Blood tests at home	95
Wound care at home	95
Self-administration of oral medications	94
House calls by therapists	90
Wearable devices for remote monitoring	90
House calls by doctors and nurses	83
Food deliveries	83
Diagnostic scans at home	71
Injections/infusions at home	70
Video consultation/ward rounds	66
Video therapies	52
**Treatment cost expectations toward home hospital, %**	
Among respondents willing to pay more	
Up to 20% more than usual care	56.5
Between 20 and 100% more than usual care	34.8
More than 200% more than usual care	8.7
Among respondents willing to pay less	
Up to 20% less than usual care	13.9
Between 20 and 50% of usual care	66.7
More than 50% less than usual care	19.5

In contrast, rejections appeared to be driven by stronger preference for care given in hospital (74% of rejecting patients), uneasiness with remote monitoring technologies (65% of rejecting patients) and a lack of confidence toward remote care delivery (58% of rejecting patients). A good proportion of these patients also wished to have nurses “in-sight” (68% of rejecting patients) and were concerned about burdening family members (46% of rejecting patients).

From the willingness-to-pay analysis, 59.3% of respondents agreed to receive home hospital care at the same price. The acceptance rate dropped to 32.8% if they would need to pay more compared to usual care. Conversely, the proportion increased to 72.2% if they could pay at least 50% less when compared to usual care.

An exploratory analysis was carried out using multivariable logistic regression to determine if there might be factors influencing the programme's acceptance (Table 3). We did not find any statistically significant associations between the dependent variable of HaH acceptance and common socioeconomic determinants including employment status, housing type, presence of domestic helper, and education level. Conversely, HaH acceptance was associated with some factors that were indirectly related to costs of care, including ward location at the point of enrolment (patients in cheaper wards being less inclined to accept the programme compared to patients in more expensive wards, *p* = 0.015), and income level (households with monthly income of < $3,000 were significantly more inclined to accept the programme, *p* = 0.04).

**Table 3:** Logistic regression model determining the association between HaH programme acceptance and selected determinants.

	**Odds ratio**	***p*-value**	**95% confidence interval**
Patient age	0.99	0.630	0.94–1.04
Patient gender			
Female	Ref		
Male	0.36	0.135	0.10–1.37
Patient race			
Non-Chinese	Ref		
Chinese	0.32	0.116	0.75–1.33
Command of selected languages			
Neither English nor Chinese	Ref		
English or Chinese	1.31	0.771	0.31–5.51
Both English and Chinese	0.79	0.815	0.10–5.92
Citizenship			
Citizen	Ref		
Permanent resident	0.21	0.138	0.03–1.66
Barthel index	0.73	0.439	0.32–1.63
Ward Location			
NUH AMU^a^	Ref		
NUH ward	0.12	0.015	0.02–0.67
AH ward	0.88	0.846	0.23–3.33
Employment status			
Unemployed	Ref		
Employed	2.55	0.165	0.68–9.57
Housing type			
Public housing	Ref		
Private housing	4.80	0.184	0.47–48.69
Marital status			
Not-currently married	Ref		
Married or domestic partnership	1.86	0.375	0.47–7.31
Presence of domestic helper			
No	Ref		
Yes	0.27	0.225	0.03–2.23
Education			
Non-tertiary and below	Ref		
Tertiary and above	2.77	0.224	0.54–14.36
Household per capita income			
Not declared	Ref		
< $3,000	5.79	0.040	1.08–31.02
≥$3,000	2.60	0.240	0.53–12.77
Self-rated overall health			
Fair and below	Ref		
Good and above	1.17	0.814	0.32–4.23

a *AMU, Acute Medical Unit was a dedicated facility within NUH that acts as the focus for acute medical care for patients that have presented as medical emergencies to hospitals. Investigations would be rapidly perform to determine patients' diagnoses and preliminary treatment plan before being discharged or transferred to other wards*.

*Model's Pseudo R2 = 0.4287*.

## Discussion

This study is the first to report attitudes and perceptions of hospitalised patients and their caregivers toward HaH care model in Asia. Our findings were contrary to our initial hypothesis that a majority of respondents would prefer care within a hospital (14, 15). In a previous qualitative study investigating care expectations among patients and caregivers in Singapore, there seemed to be an appreciable degree of reluctance among patients and family members to engage in self-care or care of their loved ones in the community compared to receiving care delivered in traditional hospitals (16). We can add an implication here e.g., this highlights the potential of introducing this care model among multi-racial Singapore society (something along these lines).

Although, we had hypothesised that demographic and socioeconomic determinants would influence patients' acceptance of HaH, many anticipated associations were not observed in the regression analysis, with the exception of some factors related to healthcare financing (namely, citizenship that determines the level of government subsidies for inpatient care, and household income). The lack of association could be because the study was neither designed nor powered to detect these differences in secondary analyses. Nonetheless, the data and findings might serve as useful baselines for the planning of future targeted studies looking into quantifiable determinants of HaH acceptance among patients and caregivers.

Our findings highlight important factors for the success of HaH that should be considered when designing such programmes: (1) At the patients' end, there should be a conducive home environment for the patients to receive treatment. Similar requirements, specifically in relation to availability of lifting equipment and lighting, had also been cited as a key factor for successful implementation overseas (17); (2) At the providers' end, there needs to be an effective coordinating centre for centralised monitoring of enrolled patients to provide timely intervention and efficient care. Such a centre can provide patients with access to care teams, enable better care management, and offer psychosocial assistance when required (18); (3) At the practice level, a multidisciplinary, competent care team can be instrumental in instilling greater confidence among patients undergoing home hospitalisation (18).

These findings also validated several crucial factors for programme planners of HaH programmes as previously reported in other studies. Firstly, support for caregivers is an important component in HaH since caregivers' willingness to take on HaH-associated responsibilities affects the ability for HaH to be implemented (2); Specific to the concern over transfer of “care burden” from hospital to home, support for caregivers is integral to ensure the well-being of caregivers and encourage their involvement in HaH. This is especially relevant for the “sandwiched generation” that experiences much social and financial pressures (19). Moving forward, it may also be important to involve community service providers in sharing the caregiving responsibility. Enlisting the help of these providers yields economies of scale and counters the loss of working hours of caregivers. Apart from financial support from government policies, community service providers may be integrated with HaH to provide assistance in activities of daily living, emotional support like counselling services and provision of round-the-clock care advice (20–22). It would be ideal for these community service providers to be reliable partners to the healthcare institutions offering HaH to co-create optimal care for the patients and their caregivers.

Secondly, effective communication between care teams and patients is crucial in enhancing patients' and caregivers' confidence toward such care model. Poor communication between healthcare professionals and patients or caregivers could amplify feelings of apprehension and distress among patients and caregivers (17); Finally, suitable adoption of devices and technologies is important to facilitate care delivery. Adequate patient/caregiver training and easy-to-use devices, such as wearables for remote vital signs monitoring, had been shown to result in greater satisfaction, improved ability to use the device and better overall functioning (18).

The high HaH programme acceptance rate among patients and family members in our study highlights an opportunity for this novel model of care. Its potential ability to tackle bed shortages is especially significant among health systems grappling with ageing populations and an increasing burden of multimorbid, complex cases. Moreover, the current COVID-19 pandemic provides greater impetus for HaH implementation. Such programmes can be pivotal in decentralising care, facilitating rapid ramp-ups in bed capacity, and controlling nosocomial infections (23, 24). For selected patients, HaH could arguably be considered better care. For instance, ED and inpatient ward environments may make delirium worse, while OT assessment in home environment is more accurate (25, 26).

In terms of programme financing and sustainability, we noted that for every 10 respondents we surveyed, six of them agreed to receive home hospital care at the same price, while two more might agree to receive home hospital care if it was 50% cheaper compared to usual care. This willingness-to-pay analysis highlights the importance of appropriate financing models to support HaH to ensure that out-of-pocket payments are at least cost-neutral to inpatient care. If HaH were cheaper than inpatient care that may encourage only a marginal increase in uptake.

Notwithstanding the potential benefits of HaH, the inherent complexity of HaH poses implementation challenges and several factors should be deliberated when planning such schemes. At a broad level, contextual factors for consideration include hospital location and resources and healthcare payment structures. Given the targeted approach of these programmes, HaH may be more practical for larger hospitals with sufficient and predictable casemix to allow for greater economies of scale (27–29).

Moreover, many countries where HaH has been widely implemented (e.g., Australia, the UK) have single-payer systems and strong imperatives to keep medical costs low, whereas, greater barriers are evident in countries such as the US where payment norms are still predominantly episodic and on a fee-for-service basis (30). Nonetheless, with the shift toward value-based payments, capitation, and integrated care across health systems, a more conducive environment for HaH may be emerging (31, 32).

From this study, we have also identified three key enablers for developing successful HaH programmes. First, patients and caregivers must be engaged and assured of sufficient support and oversight from hospital's care teams during the patients' course of care at home. Second, healthcare financing must treat home hospitalisation equally as ward hospitalisation, allowing equivalent subsidies and insurance coverage such that it is cost-neutral or cost-saving to patients who select this option. Third, careful selection and implementation of easy-to-use devices and information technology enablers are key in effective delivery of care at home. In order to validate these hypotheses, further, prospective evaluation of HaH care is warranted (33).

The study has some limitations. The study respondents were relatively young (mean age 53.6 ± 15.3) compared to the approximate median age of 68 among General Medicine patients receiving public hospital care in Singapore. While these characteristics might not represent the older patient groups with the highest healthcare demands, their views were important because such patients will represent the key target segment for HaH programmes in the future, should it get mainstreamed. Secondly, caregivers surveyed in this study excluded paid domestic help. Such domestic helpers were employed by ~25% of survey respondents in this study. This could also be a topic for future exploration. Thirdly, while there were appreciable between-group differences in the willingness-to-pay analysis, they were not reproduced when we examined surrogate markers of socio-economic status in the logistic regression. While the discrepancy between results were indeed present, it was not entirely unexpected due to the small sample size of this study and the relatively large number of variables included in the logistic regression model. Nonetheless, the exploratory findings allowed us to have an indicative understanding of factors that might or might not influence our patients' decision to participate in such programmes.

## Conclusion

From the programme acceptance rate reported in this study, HaH may have the potential to substitute substantial proportion of physical “bed-days” in compact urban Singapore. Our findings suggest that HaH programmes may be well-received even in Asia, where the perception of many toward hospital care remains entrenched within the walls of a traditional hospital.

## Data Availability Statement

The raw data supporting the conclusions of this article will be made available by the authors, without undue reservation.

## Ethics Statement

The studies involving human participants were reviewed and approved by National Healthcare Group Domain Specific Review Board (Ref: 2020/00127). The patients/participants provided their written informed consent to participate in this study.

## Author Contributions

All authors listed have made a substantial, direct and intellectual contribution to the work, and approved it for publication.

## Conflict of Interest

The authors declare that the research was conducted in the absence of any commercial or financial relationships that could be construed as a potential conflict of interest.

## Publisher's Note

All claims expressed in this article are solely those of the authors and do not necessarily represent those of their affiliated organizations, or those of the publisher, the editors and the reviewers. Any product that may be evaluated in this article, or claim that may be made by its manufacturer, is not guaranteed or endorsed by the publisher.
