# Supplementary material for: Asian Attitudes and Perceptions Toward Hospital-At-Home: A Cross-Sectional Study
Source: Front Public Health. 2021 Jul 23;9:704465. doi: 10.3389/fpubh.2021.704465 (PMC8343062; doi:10.3389/fpubh.2021.704465)
Supplement: Supplementary file 1 [file Data_Sheet_1.DOCX]

**CAREGIVER SURVEY**

**FOR CAREGIVERS OF PATIENTS WITHOUT MENTAL CAPACITY**

**PARTICIPANT INFORMATION**

| A1. | Is the patient a…? | 1. Singapore Citizen  2. Permanent Resident  (Exclude foreigner) |
| --- | --- | --- |
| A2. | Interviewer to record the patient’s ward | 1. NUH AMU  2. NUH EDTU  3. NUH ward: _____  4. AH ward: ____ |
| A3. | What is the patient’s employment status? | \| Employed full time \|  \| \| --- \| --- \| \| Employed part time \|  \| \| Self employed \|  \| \| Unemployed \|  \| \| Retired \|  \| |
| A4. | What is the patient’s occupation? | 1. Agricultural and fishery worker 2. Cleaners, labourers and related workers 3. Clerical workers 4. Legislators, senior officials and managers 5. Not employed 6. Plant/Machine operators and assemblers 7. Production craftsmen and related workers 8. Professionals 9. Service workers, shops, market sales workers 10. Technicians and associated professions 11. Others: __________________ |
| A5. | What is the patient’s residence type? | 1. Private Landed Property 2. Private Condominium 3. HDB 5 room flat/EC 4. HDB 3-4 room flat 5. HDB 1-2 room flat |
| A6. | What is the patient’s marital status? | 1. Single, never married 2. Married or domestic partnership 3. Widowed 4. Divorced or separated |
| A7. | Who does the patient live with? (select all applicable) | 🞏 Spouse  🞏 Parents  🞏 Children  🞏 Grandchildren  🞏 Grandparents  🞏 Friends  🞏 Live alone  🞏 Others: ____ |
| A8. | Is there a domestic helper present? | 🞏 Yes  🞏 No |
| A9. | What language does the patient mainly speak at home? | 1. English  2. Mandarin  3. Malay  4. Tamil  5. Dialect (Select: Teochew, Cantonese, Hokkien, Others _____)  6. Others: ____ |
| A10. | What is the patient’s highest education level achieved? | 1. No formal education  2. Primary  3. Secondary  4. A Level  5. Diploma  6. Degree and above |
| A11. | What is the patient’s per capita monthly household income? | 1. <$1000  2. $1,000-3,000  3. $3,000-$5,000  4. >$5,000  5. Prefer not to say |
| A12. | In general, how would you rate the patient’s overall health? | 1. Excellent  2. Very good  3. Good  4. Fair  5. Poor |
| A13. | The Barthel Index | Please rate what is accurate at patient’s **baseline** (usual status prior to hospitalisation). |
|  | Bowels | 0 = incontinent (or needs enema)  1 = occasional accident (once/week)  2 = continent |
|  | Bladder | 0 = incontinent, or catheterized & unable to manage  1 = occasional accident (max, once/24h)  2 = continent (for over 7 days_ |
|  | Grooming | 0 = needs help with personal care  1 = independent face/hair/teeth/shaving |
|  | Toilet Use | 0 = dependent  1 = needs some help, but can do something alone 2 = independent (on and off, dressing, wiping) |
|  | Feeding | 0 = unable  1 = needs help cutting, spreading butter, etc  2 = independent (food provided within reach) |
|  | Transfer | 0 = unable – no sitting balance  1 = major help (1-2 people), can sit  2 = minor help (verbal or physical)  3 = independent |
|  | Mobility | 0 = immobile  1 = wheelchair independent, including corners, etc  2 = walks with help of one person (verbal or physical)  3 = independent (but may use any aid, e.g. stick) |
|  | Dressing | 0 = dependent  1 = needs help, but can do half unaided  2 = independent |
|  | Stairs | 0 = unable  1 = needs help (verbal, physical)  2 = independent up and down |
|  | Bathing | 0=independent  1= independent |
| A14. | Rate the patients’ independence in carrying out daily activities. | \|  \| Independent \| Needs help \| Unable \| \| --- \| --- \| --- \| --- \| \| Taking medications \|  \|  \|  \| \| Grocery shopping \|  \|  \|  \| \| Preparing meals \|  \|  \|  \| \| Using the telephone \|  \|  \|  \| \| Taking public transportation \|  \|  \|  \| \| Handling own finances \|  \|  \|  \| \| Housekeeping \|  \|  \|  \| \| Laundry \|  \|  \|  \| \| Going to the doctor \|  \|  \|  \| |
| A15. | If the patient requires assistance, who is the main person who assists you at home? | Actual person____  Interviewer to code: caregiver is patient’s  1. Spouse  2. Parent  3. Child  4. Other relative  5. Unrelated person  6. Domestic helper |

**CAREGIVER INFORMATION**

The following questions relate to you, the main caregiver, and not the patient

| B1. | Are you the main caregiver?  If no, who else provide care to the patient? | 1. Yes  2. No  Answer: |
| --- | --- | --- |
| B2. | What is your age? | Actual age:______ |
| B3. | What is your highest education level achieved? | 1. No formal education  2. Primary  3. Secondary  4. A Level  5. Diploma  6. Degree and above |
| B4. | What language do you mainly speak at home? | 1. English  2. Mandarin  3. Malay  4. Tamil  5. Dialect (Select: Teochew, Cantonese, Hokkien, Others _____)  6. Others: ____ |
| B5. | What is your relationship with the patient? | Actual relationship____  Interviewer to code: caregiver is patient’s  1. Spouse  2. Parent  3. Child  4. Grandchild  5. Grandparent  6. Friend  7. Others: ____ |

**CAREGIVER-RATED Health-Related Quality of Life**

Under each heading, please tick the ONE box that best describes how you would rate the patient’s health TODAY.

| C1. | MOBILITY   I have no problems in walking about   I have slight problems in walking about   I have moderate problems in walking about   I have severe problems in walking about   I am unable to walk about |
| --- | --- |
| C2. | SELF-CARE   I have no problems in washing or dressing myself   I have slight problems in washing or dressing myself   I have moderate problems in washing or dressing myself   I have severe problems in washing or dressing myself   I am unable to wash or dress myself |
| C3. | USUAL ACTIVITIES (e.g. work, study, housework, family or leisure activities)   I have no problems doing my usual activities   I have slight problems in doing my usual activities   I have moderate problems in doing my usual activities   I have severe problems in doing my usual activities   I am unable to do my usual activities |
| C4. | PAIN/DISCOMFORT   I have no pain or discomfort   I have slight pain or discomfort   I have moderate pain or discomfort   I have severe pain or discomfort   I have extreme pain or discomfort |
| C5. | ANXIETY/DEPRESSION   I am not anxious or depressed   I am slightly anxious or depressed   I am moderately anxious or depressed   I am severely anxious or depressed   I am extremely anxious or depressed |
| C6. | 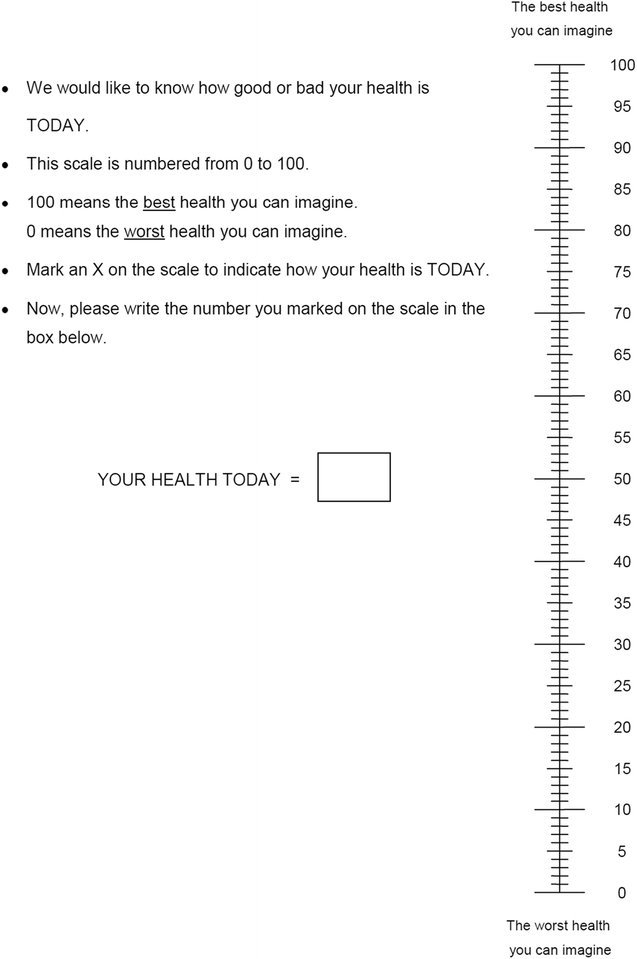 |

**CARE PROVIDED IN HOSPITAL**

These questions relate to the patient experience in the ward so far.

| D1. | How many times (excluding this admission) has the patient been admitted to hospital in the last 12 months? | [number] |
| --- | --- | --- |
| D2. | Based on your knowledge, which persons cared for the patient since being admitted to hospital? Select all that apply. | 🞏 Doctors  🞏 Nurses  🞏 Physical therapists  🞏 Pharmacist  🞏 Social Worker  🞏 Dietician  🞏 Others ________ |
| D3. | Which procedures have been done for the patient since admission to hospital? Select all that apply. | 🞏 Taking blood pressure  🞏 Having blood tests  🞏 Having physiotherapy  🞏 Going for scans  🞏 Having wounds dressed  🞏 Served tablet medication  🞏 Given injection medication (drip)  🞏 Counselling  🞏 Others ________ |
| D4. | How does the patient pass urine/motion at present? | 1. Walk to toilet independently  2. Walk to toilet with assistance  3. Commode independently  4. Commode with assistance  5. Diapers  6. Catheter/Stoma/Other devices |

**VIEWS ABOUT CARE PROVIDED IN AT HOME**

We are developing a programme that can take care of hospitalized patients in their own homes rather than in hospital. We would send doctors, nurses and therapists to the home and set up IVs and take blood tests in your home instead, in a “temporary hospital unit”. For the next questions, we will be asking you about how you would feel about this.

| E1. | If all of this care can be provided at the patient’s home instead with a “temporary hospital unit” set up, would you agree for the patient to participate? | 1. Yes  2. No |
| --- | --- | --- |
| E2. | I would be comfortable if the following healthcare professionals visited the patient at home.  SA: Strongly Agree  A: Agree  D: Disagree  SD: Strong disagree | \|  \| SA \| A \| D \| SD \| \| --- \| --- \| --- \| --- \| --- \| \| Doctors and nurses coming to visit the patient at home daily \|  \|  \|  \|  \| \| Physiotherapist helping the patient exercise at home \|  \|  \|  \|  \| \| Receiving food delivery to my home \|  \|  \|  \|  \| |
| E3. | I would be comfortable if the following treatment was done for the patient at home rather than in the hospital.  SA: Strongly Agree  A: Agree  D: Disagree  SD: Strong disagree | \|  \| SA \| A \| D \| SD \| \| --- \| --- \| --- \| --- \| --- \| \| Nurses taking blood tests at home \|  \|  \|  \|  \| \| Nursing doing wound dressing at home \|  \|  \|  \|  \| \| Taking tablets from a pre-packed pill box at home rather than having a nurse serve the patient pills \|  \|  \|  \|  \| \| IV drip at home \|  \|  \|  \|  \| \| Scans done at home rather than in the hospital \|  \|  \|  \|  \| \| Transfer to hospital if further scans are needed \|  \|  \|  \|  \| |
| E4. | I would be comfortable if the following technology was used to help the patient to be cared for at home rather than in the hospital.  SA: Strongly Agree  A: Agree  D: Disagree  SD: Strong disagree | \|  \| SA \| A \| D \| SD \| \| --- \| --- \| --- \| --- \| --- \| \| Wearing a patch or watch to monitor blood pressure and heart rate continuously rather than blood pressure being taken by nurses every few hours \|  \|  \|  \|  \| \| Talking to doctors and nurses through video call rather than having them come to see the patient at the bedside, when appropriate \|  \|  \|  \|  \| \| Exercising by video call with a physiotherapist \|  \|  \|  \|  \| |
| E5. | How much do you agree with the following statements with regards to implementing hospital care at home?  SA: Strongly Agree  A: Agree  D: Disagree  SD: Strong disagree | \|  \| SA \| A \| D \| SD \| \| --- \| --- \| --- \| --- \| --- \| \| The patient may fall at home \|  \|  \|  \|  \| \| I feel more comfortable having the patient at home than in hospital \|  \|  \|  \|  \| \| It will be too expensive \|  \|  \|  \|  \| \| I will be inconvenienced \|  \|  \|  \|  \| \| I prefer for the patient to be admitted to hospital rather than being at home when unwell \|  \|  \|  \|  \| \| Having nurses available within sight is important to me \|  \|  \|  \|  \| \| I prefer the patient having family around at home. \|  \|  \|  \|  \| \| I would feel unsafe if the patient was at home. \|  \|  \|  \|  \| \| It would be important to me to have the patients vital signs monitored continuously if they were cared for at home. \|  \|  \|  \|  \| |
| E6. | How confident are you that the patient’s condition can be adequately treated with the services at home? | 1. Very confident  2. Somewhat confident  3. Not very confident  4. Not confident at all |
| E7. | If the patient were to be offered this “hospital at home” programme, would you agree? | 1. Definitely yes  2. Probably yes  3. Probably no  4. Definitely no |
| E8. | Why or why not? | [free text answer] |
| E9. | What are some advantages of “hospital at home” care? | [free text answer] |
| E10. | What are some problems in “hospital at home” care? | [free text answer] |
| E11. | Any other comments? | [free text answer] |

**VIEWS ABOUT PAYMENT**

The next section will explore how much you are willing to pay (or not) for such a service.

| F1. | If the patient were to be offered this “hospital at home” programme at same cost to being in a ward (covered by medisave and insurance), would you agree? | | 1. Definitely yes (proceed to F2)  2. Probably yes (proceed to F2)  3. Probably no (proceed to F4)  4. Definitely no (proceed to F4) |
| --- | --- | --- | --- |
| F2. | If the patient were to be offered this “hospital at home” programme at **higher** cost than being in a ward, would you agree for the patient to participate? | | 1. Definitely yes  2. Probably yes  3. Probably no 🡪 skip F3, F4, F5  4. Definitely no 🡪 skip F3, F4, F5 |
| F3. | Suppose that one day of hospital stay cost $100 per day after all subsidies including medisave.  Please fill in the blank: “I would prefer the patient to stay at home and receive care through the hospital-at-home program even if the cost per day was $___ “ | | [free text in $] 🡪 skip F4 and F5  (note to interviewer: this cost must be > 100) |
| F4. | | If you were to be offered this “hospital at home” programme at **lower** cost than being in a ward, would you agree for the patient to participate? | 1. Definitely yes  2. Probably yes  3. Probably no 🡪 Skip F5  4. Definitely no 🡪 Skip F5 |
| F5. | | Suppose that one day of hospital stay cost $100 per day after all subsidies including medisave.  Please fill in the blank: “I would prefer the patient to stay at home and receive care through the hospital-at-home program if the cost per day was $___ “ | [free text in $]  (note to interviewer: this cost must be < 100) |

**CLOSING**

Thank you so much for participating today.

| G1. | Would you be okay if we had access to view the patient’s medical records to better understand their condition? | 1. Yes  2. No |
| --- | --- | --- |
| G2. | ID |  |

*If caregiver and patient are doing the survey together, only sections B, D, E, F, and G should be repeated for the caregiver

Thank you once again for your time today.

Paper data collection:

| A. | ID (linked to survey ID) |  |
| --- | --- | --- |
| B. | Patient NRIC |  |
| C. | Contact number for interview |  |

For patients who consent to G2, to access CPSS records to obtain

- Age
- Gender
- Race
- Ward class
- Primary diagnosis
- Length of stay
- Eligibility for HAH
