# Supplementary material for: Asian Attitudes and Perceptions Toward Hospital-At-Home: A Cross-Sectional Study
Source: Front Public Health. 2021 Jul 23;9:704465. doi: 10.3389/fpubh.2021.704465 (PMC8343062; doi:10.3389/fpubh.2021.704465)
Supplement: Supplementary file 2 [file Data_Sheet_2.DOCX]

**PATIENT SURVEY**

**FOR PATIENTS WHO HAVE MENTAL CAPACITY**

**PARTICIPANT INFORMATION**

| A1. | Are you a…? | 1. Singapore Citizen  2. Permanent Resident  (Exclude foreigner) |
| --- | --- | --- |
| A2. | Interviewer to record the patient’s ward | 1. NUH AMU  2. NUH EDTU  3. NUH ward: _____  4. AH ward: ____ |
| A3. | Employment status | \| Employed full time \|  \| \| --- \| --- \| \| Employed part time \|  \| \| Self employed \|  \| \| Unemployed \|  \| \| Retired \|  \| |
| A4. | What is your occupation? | 1. Agricultural and fishery worker 2. Cleaners, labourers and related workers 3. Clerical workers 4. Legislators, senior officials and managers 5. Not employed 6. Plant/Machine operators and assemblers 7. Production craftsmen and related workers 8. Professionals 9. Service workers, shops, market sales workers 10. Technicians and associated professions 11. Others: __________________ |
| A5. | What is your residence type? | 1. Private Landed Property 2. Private Condominium 3. HDB 5 room flat/EC 4. HDB 3-4 room flat 5. HDB 1-2 room flat |
| A6. | What is your marital status? | 1. Single, never married 2. Married or domestic partnership 3. Widowed 4. Divorced or separated |
| A7. | Who do you live with? (select all applicable) | 🞏 Spouse  🞏 Parents  🞏 Children  🞏 Grandchildren  🞏 Grandparents  🞏 Friends  🞏 Live alone  🞏 Others: ____ |
| A8. | Is there a domestic helper present? | 🞏 Yes  🞏 No |
| A9. | What language do you mainly speak at home? | 1. English  2. Mandarin  3. Malay  4. Tamil  5. Dialect (Select: Teochew, Cantonese, Hokkien, Others _____)  6. Others: ____ |
| A10. | What is your highest education level achieved? | 1. No formal education  2. Primary  3. Secondary  4. A Level  5. Diploma  6. Degree and above |
| A11. | What is your monthly per capita household income? | 1. <$1000  2. $1,000-3,000  3. $3,000-$5,000  4. >$5,000  5. Prefer not to say |
| A12. | In general, how would you rate your overall health? | 1. Excellent  2. Very good  3. Good  4. Fair  5. Poor |
| A13. | The Barthel Index | Please rate what is accurate at your **baseline** (usual status prior to hospitalisation). |
|  | Bowels | 0 = incontinent (or needs enema)  1 = occasional accident (once/week)  2 = continent |
|  | Bladder | 0 = incontinent, or catheterized & unable to manage  1 = occasional accident (max, once/24h)  2 = continent (for over 7 days_ |
|  | Grooming | 0 = needs help with personal care  1 = independent face/hair/teeth/shaving |
|  | Toilet Use | 0 = dependent  1 = needs some help, but can do something alone 2 = independent (on and off, dressing, wiping) |
|  | Feeding | 0 = unable  1 = needs help cutting, spreading butter, etc  2 = independent (food provided within reach) |
|  | Transfer | 0 = unable – no sitting balance  1 = major help (1-2 people), can sit  2 = minor help (verbal or physical)  3 = independent |
|  | Mobility | 0 = immobile  1 = wheelchair independent, including corners, etc  2 = walks with help of one person (verbal or physical)  3 = independent (but may use any aid, e.g. stick) |
|  | Dressing | 0 = dependent  1 = needs help, but can do half unaided  2 = independent |
|  | Stairs | 0 = unable  1 = needs help (verbal, physical)  2 = independent up and down |
|  | Bathing | 0=independent  1= independent |
| A14. | Rate your independence in carrying out daily activities. | \|  \| Independent \| Needs help \| Unable \| \| --- \| --- \| --- \| --- \| \| Taking medications \|  \|  \|  \| \| Grocery shopping \|  \|  \|  \| \| Preparing meals \|  \|  \|  \| \| Using the telephone \|  \|  \|  \| \| Taking public transportation \|  \|  \|  \| \| Handling own finances \|  \|  \|  \| \| Housekeeping \|  \|  \|  \| \| Laundry \|  \|  \|  \| \| Going to the doctor \|  \|  \|  \| |
| A15. | If you require assistance, who is the main person who assists you at home? | Actual person____  Interviewer to code: caregiver is patient’s  1. Spouse  2. Parent  3. Child  4. Other relative  5. Unrelated person  6. Domestic helper |

**LITERACY AND COGNITION**

| B1. | Mini-Cog | 1. Pass (no cognitive impairment) 2. Fail (cognitive impairment) |
| --- | --- | --- |
| B2. | How often do you have problems learning about your medical condition because of difficulty understanding written information? | 1. Never 2. Occasionally 3. Sometimes 4. Often 5. Always |
| B3. | How confident are you filling out medical forms by yourself? | 1. Extremely 2. Quite a bit 3. Somewhat 4. A little bit 5. Not at all |
| B4. | How often do you have someone help you read hospital materials? | 1. Never 2. Occasionally 3. Sometimes 4. Often 5. Always |

**
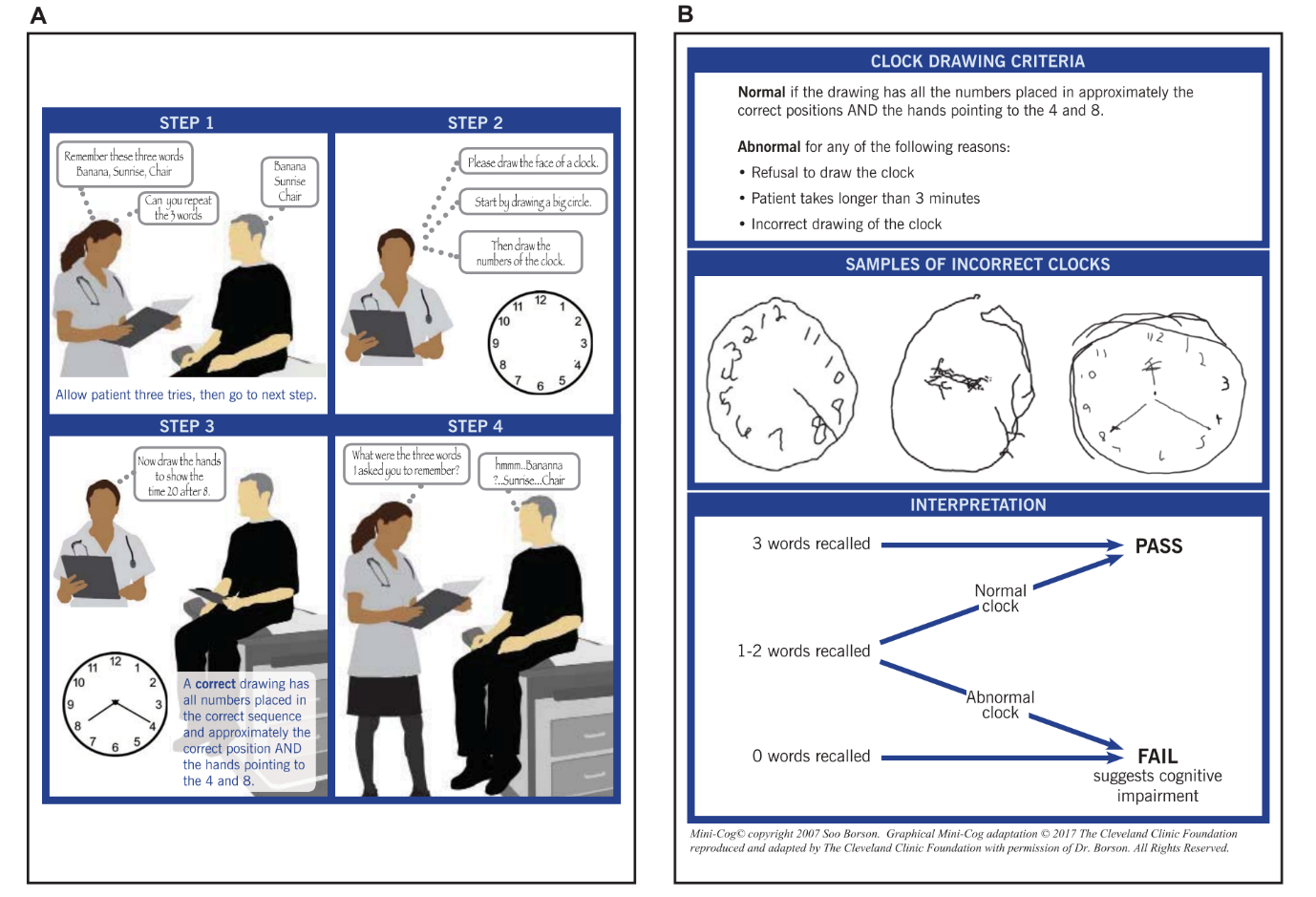
**

**PATIENT-RATED Health-Related Quality of Life**

Under each heading, please tick the ONE box that best describes your health TODAY.

| C1. | MOBILITY   I have no problems in walking about   I have slight problems in walking about   I have moderate problems in walking about   I have severe problems in walking about   I am unable to walk about |
| --- | --- |
| C2. | SELF-CARE   I have no problems in washing or dressing myself   I have slight problems in washing or dressing myself   I have moderate problems in washing or dressing myself   I have severe problems in washing or dressing myself   I am unable to wash or dress myself |
| C3. | USUAL ACTIVITIES (e.g. work, study, housework, family or leisure activities)   I have no problems doing my usual activities   I have slight problems in doing my usual activities   I have moderate problems in doing my usual activities   I have severe problems in doing my usual activities   I am unable to do my usual activities |
| C4. | PAIN/DISCOMFORT   I have no pain or discomfort   I have slight pain or discomfort   I have moderate pain or discomfort   I have severe pain or discomfort   I have extreme pain or discomfort |
| C5. | ANXIETY/DEPRESSION   I am not anxious or depressed   I am slightly anxious or depressed   I am moderately anxious or depressed   I am severely anxious or depressed   I am extremely anxious or depressed |
| C6. | 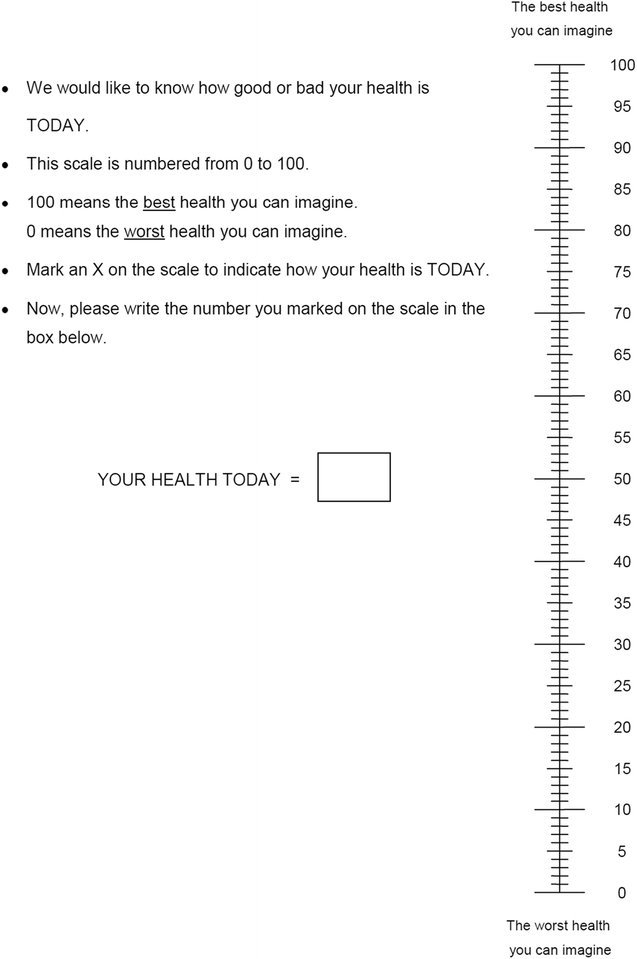 |

**CARE PROVIDED IN HOSPITAL**

These questions relate to your experience in the ward so far.

| D1. | How many times (excluding this admission) have you been admitted to hospital in the last 12 months? | [number] |
| --- | --- | --- |
| D2. | Which persons cared for you since being admitted to hospital? Select all that apply. | 🞏 Doctors  🞏 Nurses  🞏 Physical therapists  🞏 Pharmacist  🞏 Social Worker  🞏 Dietician  🞏 Others ________ |
| D3. | Which procedures have been done for you since admission to hospital? Select all that apply. | 🞏 Taking blood pressure  🞏 Having blood tests  🞏 Having physiotherapy  🞏 Going for scans  🞏 Having wounds dressed  🞏 Served tablet medication  🞏 Given injection medication (drip)  🞏 Counselling  🞏 Others ________ |
| D4. | Now that you are in hospital, how do you pass urine/motion at present? | 1. Walk to toilet independently  2. Walk to toilet with assistance  3. Commode independently  4. Commode with assistance  5. Diapers  6. Catheter/Stoma/Other devices |

**VIEWS ABOUT CARE PROVIDED IN AT HOME**

We are developing a programme that can take care of hospitalized patients in their own homes rather than in hospital. We would send doctors, nurses and therapists to the home and set up IVs and take blood tests in your home instead, in a “temporary hospital unit”. For the next questions, we will be asking you about how you would feel about this.

| E1. | If all of this care can be provided at home instead with a “temporary hospital unit” set up at your home, would you agree to participate? | 1. Yes  2. No |
| --- | --- | --- |
| E2. | I would be comfortable if the following healthcare professionals visited me at home.  SA: Strongly Agree  A: Agree  D: Disagree  SD: Strong disagree | \|  \| SA \| A \| D \| SD \| \| --- \| --- \| --- \| --- \| --- \| \| Doctors and nurses coming to visit me at home daily \|  \|  \|  \|  \| \| Physiotherapist helping me exercise at my home \|  \|  \|  \|  \| \| Receiving food delivery to my home \|  \|  \|  \|  \| |
| E3. | I would be comfortable if the following treatment was done at home rather than in the hospital.  SA: Strongly Agree  A: Agree  D: Disagree  SD: Strong disagree | \|  \| SA \| A \| D \| SD \| \| --- \| --- \| --- \| --- \| --- \| \| Nurses taking my blood tests at home \|  \|  \|  \|  \| \| Nursing doing wound dressing at home \|  \|  \|  \|  \| \| Taking tablets from a pre-packed pill box at home rather than having a nurse serve me my pills \|  \|  \|  \|  \| \| IV drip at home \|  \|  \|  \|  \| \| Scans done at home rather than in the hospital \|  \|  \|  \|  \| \| Transfer to hospital if further scans are needed \|  \|  \|  \|  \| |
| E4. | I would be comfortable if the following technology was used to help me be cared for at home rather than in the hospital.  SA: Strongly Agree  A: Agree  D: Disagree  SD: Strong disagree | \|  \| SA \| A \| D \| SD \| \| --- \| --- \| --- \| --- \| --- \| \| Wearing a patch or watch to monitor my blood pressure and heart rate continuously rather than blood pressure being taken by nurses every few hours \|  \|  \|  \|  \| \| Talking to doctors and nurses through video call rather than having them come to see me at the bedside, when appropriate \|  \|  \|  \|  \| \| Exercising by video call with a physiotherapist \|  \|  \|  \|  \| |
| E5. | How much do you agree with the following statements with regards to implementing hospital care at home?  SA: Strongly Agree  A: Agree  D: Disagree  SD: Strong disagree | \|  \| SA \| A \| D \| SD \| \| --- \| --- \| --- \| --- \| --- \| \| I may fall at home \|  \|  \|  \|  \| \| I feel more comfortable at home than in hospital \|  \|  \|  \|  \| \| I will inconvenience my family members \|  \|  \|  \|  \| \| I prefer being admitted to hospital than being at home when I am unwell \|  \|  \|  \|  \| \| Having nurses available within sight is important to me \|  \|  \|  \|  \| \| I prefer having my family around me at home. \|  \|  \|  \|  \| \| I would feel unsafe at home. \|  \|  \|  \|  \| \| It would be important to me to have my vital signs monitored continuously if I were cared for at home. \|  \|  \|  \|  \| |
| E6. | How confident are you that your condition can be adequately treated with the services at home? | 1. Very confident  2. Somewhat confident  3. Not very confident  4. Not confident at all |
| E7. | If you were to be offered this “hospital at home” programme, would you agree? | 1. Definitely yes  2. Probably yes  3. Probably no  4. Definitely no |
| E8. | Why or why not? | [free text answer] |
| E9. | What are some advantages of “hospital at home” care? | [free text answer] |
| E10. | What are some problems in “hospital at home” care? | [free text answer] |
| E11. | Any other comments? | [free text answer] |

**VIEWS ABOUT PAYMENT**

The next section will explore how much you are willing to pay (or not) for such a service.

| F1. | If you were to be offered this “hospital at home” programme at same cost to being in a ward (covered by medisave and insurance), would you agree? | | 1. Definitely yes (proceed to F2)  2. Probably yes (proceed to F2)  3. Probably no (proceed to F4)  4. Definitely no (proceed to F4) |
| --- | --- | --- | --- |
| F2. | If you were to be offered this “hospital at home” programme at **higher** cost than being in a ward, would you agree to participate? | | 1. Definitely yes  2. Probably yes  3. Probably no 🡪 skip F3, F4, F5  4. Definitely no 🡪 skip F3, F4, F5 |
| F3. | Suppose that one day of hospital stay cost you $100 per day after all subsidies including medisave.  Please fill in the blank: “I would prefer to stay at home and receive care through the hospital-at-home program even if the cost per day was $___ “ | | [free text in $] 🡪 skip F4 and F5  (note to interviewer: this cost must be > 100) |
| F4. | | If you were to be offered this “hospital at home” programme at **lower** cost than being in a ward, would you agree to participate? | 1. Definitely yes  2. Probably yes  3. Probably no 🡪 Skip F5  4. Definitely no 🡪 Skip F5 |
| F5. | | Suppose that one day of hospital stay cost you $100 per day after all subsidies including medisave.  Please fill in the blank: “I would prefer to stay at home and receive care through the hospital-at-home program if the cost per day was $___ “ | [free text in $]  (note to interviewer: this cost must be < 100) |

**CLOSING**

Thank you so much for participating today.

| G1. | Would you be okay if we had access to view your medical records to better understand your condition? | 1. Yes  2. No |
| --- | --- | --- |
| G2. | ID |  |

Thank you once again for your time today.

Paper data collection:

| A. | ID (linked to survey ID) |  |
| --- | --- | --- |
| B. | NRIC |  |
| C. | Contact number for interview |  |

For patients who consent to G2, to access CPSS records to obtain

- Age
- Gender
- Race
- Ward class
- Primary diagnosis
- Length of stay
- Eligibility for HAH
